# Calling in the Cold: Pervasive Acoustic Presence of Humpback Whales (*Megaptera novaeangliae*) in Antarctic Coastal Waters

**DOI:** 10.1371/journal.pone.0073007

**Published:** 2013-09-06

**Authors:** Ilse Van Opzeeland, Sofie Van Parijs, Lars Kindermann, Elke Burkhardt, Olaf Boebel

**Affiliations:** 1 Ocean Acoustics Lab, Alfred Wegener Institute for Polar and Marine Research, Bremerhaven, Germany; 2 Northeast Fisheries Science Center, Woods Hole, Massachusetts, United States of America; Università degli Studi di Napoli Federico II, Italy

## Abstract

Humpback whales migrate between relatively unproductive tropical or temperate breeding grounds and productive high latitude feeding areas. However, not all individuals of a population undertake the annual migration to the breeding grounds; instead some are thought to remain on the feeding grounds year-round, presumably to avoid the energetic demands of migration. In the Southern Hemisphere, ice and inclement weather conditions restrict investigations of humpback whale presence on feeding grounds as well as the extent of their southern range. Two years of near-continuous recordings from the PerenniAL Acoustic Observatory in the Antarctic Ocean (PALAOA, Ekström Iceshelf, 70°31’S, 8°13’W) are used to explore the acoustic presence of humpback whales in an Antarctic coastal area. Humpback whale calls were present during nine and eleven months of 2008 and 2009, respectively. In 2008, calls were present in January through April, June through August, November and December, whereas in 2009, calls were present throughout the year, except in September. Calls occurred in un-patterned sequences, representing non-song sound production. Typically, calls occurred in bouts, ranging from 2 to 42 consecutive days with February, March and April having the highest daily occurrence of calls in 2008. In 2009, February, March, April and May had the highest daily occurrence of calls. Whales were estimated to be within a 100 km radius off PALAOA. Calls were also present during austral winter when ice cover within this radius was >90%. These results demonstrate that coastal areas near the Antarctic continent are likely of greater importance to humpback whales than previously assumed, presumably providing food resources year-round and open water in winter where animals can breathe.

## Introduction

Baleen whales undertake annual migrations between tropical or temperate wintering areas where breeding takes place, and high latitude feeding grounds in summer [1]. In contrast to migrations undertaken by terrestrial species, which are primarily driven by nutritional resources at both locations [2], [3], baleen whales migrate between relatively unproductive breeding grounds and productive feeding areas. Corkeron and Conner [4] revisited several hypotheses as to why baleen whales undertake these long-distance migrations and concluded that the most likely hypotheses driving baleen whale migration are those related to calf growth and survival, *i.e.,* the benefits of calm water and absence of major predators such as killer whales in the wintering areas. However, there is increasing evidence for baleen whale species, such as blue (*Balaenoptera musculus*), fin (*B. physalus*) and minke whales (*B. acutorostrata*), that not all individuals of a population undertake the annual migration and that part of the population is present on the feeding grounds during the winter [5], [6], [7], [8]. Humpback whales (*Megaptera novaeangliae*) have been shown to be present on the feeding grounds off the Alaskan coast year-round [9]. Brown *et al.,* [10] found that the sex-ratio of humpback whales migrating from the feeding grounds in the Antarctic to the breeding areas near the east-Australian coast was highly skewed towards males, demonstrating that some females remain in the feeding area year-round. This study provides clear evidence to support that humpback whales are present on the feeding grounds in winter.

Based on mark-recapture and historic catch data, most humpback whale feeding grounds in the Southern Hemisphere are believed to be located around 60°S [11], [12]. However, this knowledge is primarily based on data collected off-shore during the austral summer and is limited in scope because of heavy ice conditions close to the continent as well as limited daylight and extensive ice cover in austral winter. Consequently, the extent of the southern range of humpback whale feeding areas is unknown. In the Northern Hemisphere, humpback whales show a strong affinity for coastal waters [13], which during the summer is thought to reflect the distribution of prey species [14]. Similarly, off the western Antarctic Peninsula, resource sites for humpback whales are mainly located in near-coastal areas [15], [16], raising the question as to whether humpback whales off the Antarctic mainland might feed close to the ice-shelf edge. During Antarctic winter, vast areas of the Southern Ocean are ice covered while coastal polynyas within the ice-shelf edge region provide small areas of open water where minke whales and beaked whales have been observed [6]. Humpback whales are thought to avoid entering ice-covered areas [16], leaving the question as to whether humpback whales remain on the Antarctic feeding grounds during winter unresolved.

Humpback whales are highly vocal and are known to produce sound on the breeding and feeding grounds as well as during migration [17], [18], [19]. Passive acoustic recording techniques therefore offer a suitable tool for monitoring humpback whale presence year-round in – from the human perspective – remote areas such as the Antarctic. In this study we investigate the year-round acoustic presence of humpback whales close to the ice-shelf edge using two years of continuous passive acoustic recordings obtained by the autonomous PerenniAL Acoustic Observatory in the Antarctic Ocean (PALAOA).

## Methods

### Acoustic Recordings

**Figure 1:**
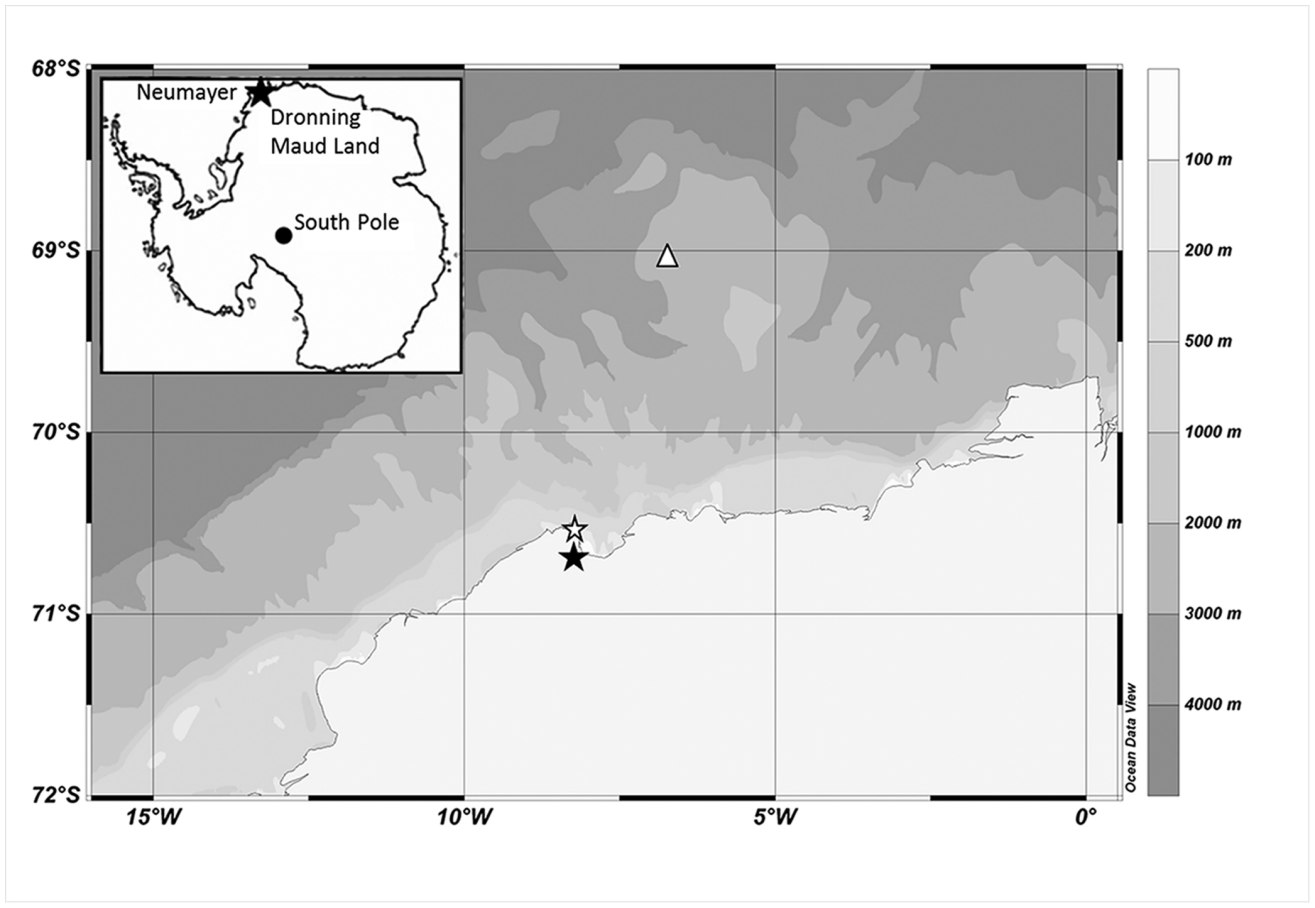
Bathymetry map [53] showing the location of PALAOA (white star). The location of the German Neumayer Station II is indicated by the black star. The white triangle indicates the location of a RAFOS sound source. Inset image: map of Antarctica showing the location of Neumayer Station (black star).

The PerenniAL Acoustic Observatory in the Antarctic Ocean (PALAOA) is located at 70°31′S, 8°13′W on the Eckström Iceshelf, eastern Weddell Sea coast, Antarctica (Fig. 1). For this study we used recordings from 2008 [20] and 2009 [21] made with a RESON TC4032 hydrophone deployed at 170 m depth underneath the 100 m thick floating Antarctic ice shelf through a bore-hole. Distance to the ice shelf edge is ∼1 km and water depth around 250 m [22]. Signals were digitized at 48 kHz/16 bit, encoded to a 192 kBit MP3 stream and stored as a sequence of time-stamped files. The effective bandwidth of the PALAOA recordings is 10 Hz to 15 kHz, the dynamic range ∼90 dB, with full scale set manually to values between 138 and 150 dB_FS_ re 1 µPa to avoid clipping in case of loud conditions. Full scale settings were logged for each file and accounted for in all calculations of sound pressure levels referred to hereinafter. PALAOA records continuously year-round, however occasional gaps occur due to power outages. For 2008 and 2009, recordings covered 72% and 91% of the year, respectively.

### Automated Call Detection

**Figure 2:**
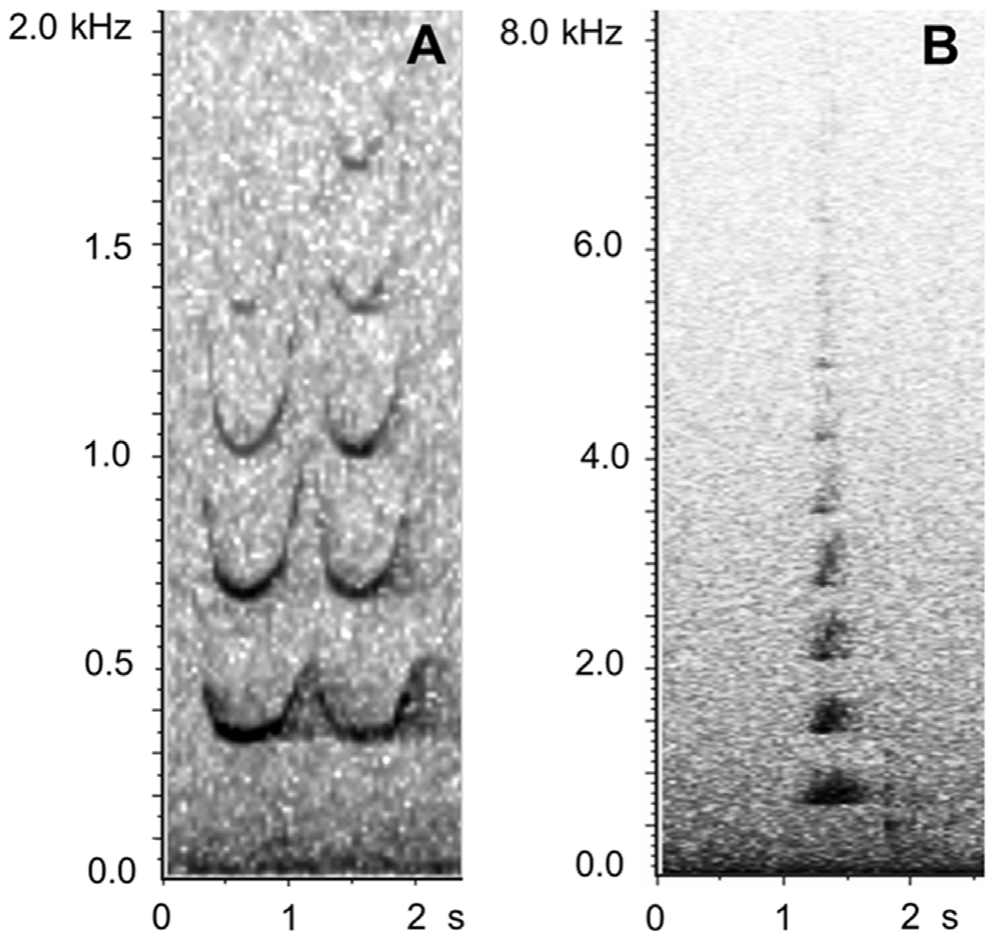
Spectrograms of two humpback whale high calls recorded by PALAOA. Spectrogram a) shows a typical double call consisting of two roughly identical elements, whereas b) represents a high call type consisting of one single call element.

**Figure 3:**
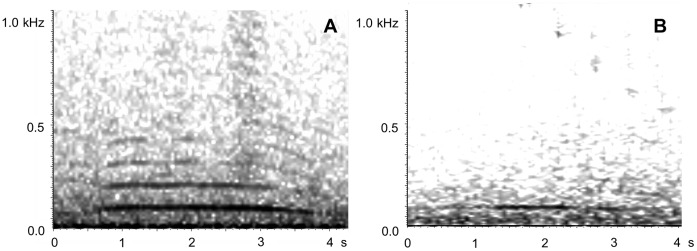
Spectrograms of humpback whale moans recorded by PALAOA. Moans recorded: a) on 1 April 2008 representing a moan from austral fall, and b) on 6 July 2008, representing a moan from austral winter.

Previous analyses showed that two predominant humpback whale vocalization types were present in the PALAOA recordings: moans and high calls [23]. Both vocalization types were positively assigned to humpback whales based on previous evidence [18], [19], [24]. Both moans and high calls were produced in un-patterned sequences, representing non-song sound production. High calls (Fig. 2, [23]) were too variable to be useful for our automated detection method and were not included in the analysis in this study. Moans are low frequency (fundamental frequency ∼100 Hz, [24]) arch-shaped calls that are sufficiently stereotyped to allow for automated detection (Fig. 3a & b). Moans were automatically detected using the ‘data template detector’ in XBAT (Bioacoustics Research Program, Cornell Lab of Ornithology, www.xbat.org, [25]). Detections are based on acoustic similarity between a specified template and the acoustic events in the recording, quantified through spectrogram cross-correlation. Detections were run with one template of the moan (duration 1.1 s, bandwidth 240 Hz, detection correlation threshold 0.42, spectrogram parameters used for template development: FFT size 4048 points, data window overlap 25%, window length = 1 (i.e., equal to the FFT size), window function = Hanning). All automated detections were visually and aurally reviewed and classified as false or true. Only verified detections were used to explore seasonal acoustic presence of humpback whales.

### Ice Cover and Acoustic Ranging

To compare humpback whale acoustic presence to local ice cover, percentage open water was calculated using ENVISAT ice cover data with a 6.25×6.25 km resolution [26]. The radius of the area off PALAOA for which percentage open water was calculated was based on estimations of the approximate distance of calling humpback whales recorded by PALAOA. This distance was estimated by comparing the received levels at PALAOA of humpback whale moans with signals of an oceanographic RAFOS sound source with comparable acoustic propagation characteristics as humpback whale moans. The RAFOS sound source was located 177 km north of PALAOA (Fig. 1). From March 2008 to December 2010, the oceanographic sound source produced a 80 s frequency modulated sweep between 259 and 261 Hz at 180 dB_rms_ re 1 µPa @ 1 m, comparable to the frequencies of humpback whale moans. RAFOS signals (N = 939) were received at levels of 70±7 dB_rms_ re 1 µPa rms, resulting in direct empirical evidence of an almost spherical transmission loss law with TL = 20.9±1.3·log_10_(r). In further calculation, the ±7 dB_rms_ standard deviation of received RAFOS levels was taken into account as the ±1.3 uncertainty in the transmission loss law. This relation was used to estimate the distances of moaning humpback whales (RL = 86±7 dB_rms_ re 1 µPa rms; N = 9), assuming moan source levels SL = 175 dB_0-p_ re 1 µPa @ 1 m [24]. Resulting distance estimates ranged between 2 and 78 km, including any variations resulting from the uncertainty in the transmission loss law.

## Results

### Seasonal Presence

For September 2008 and June 2009 acoustic data were collected on only 8 and 1 days, respectively, as PALAOA was offline due to energy shortage during that time. For 16 months recording coverage was >80%, for the remaining 6 months recordings covered ≥50%.

**Figure 4:**
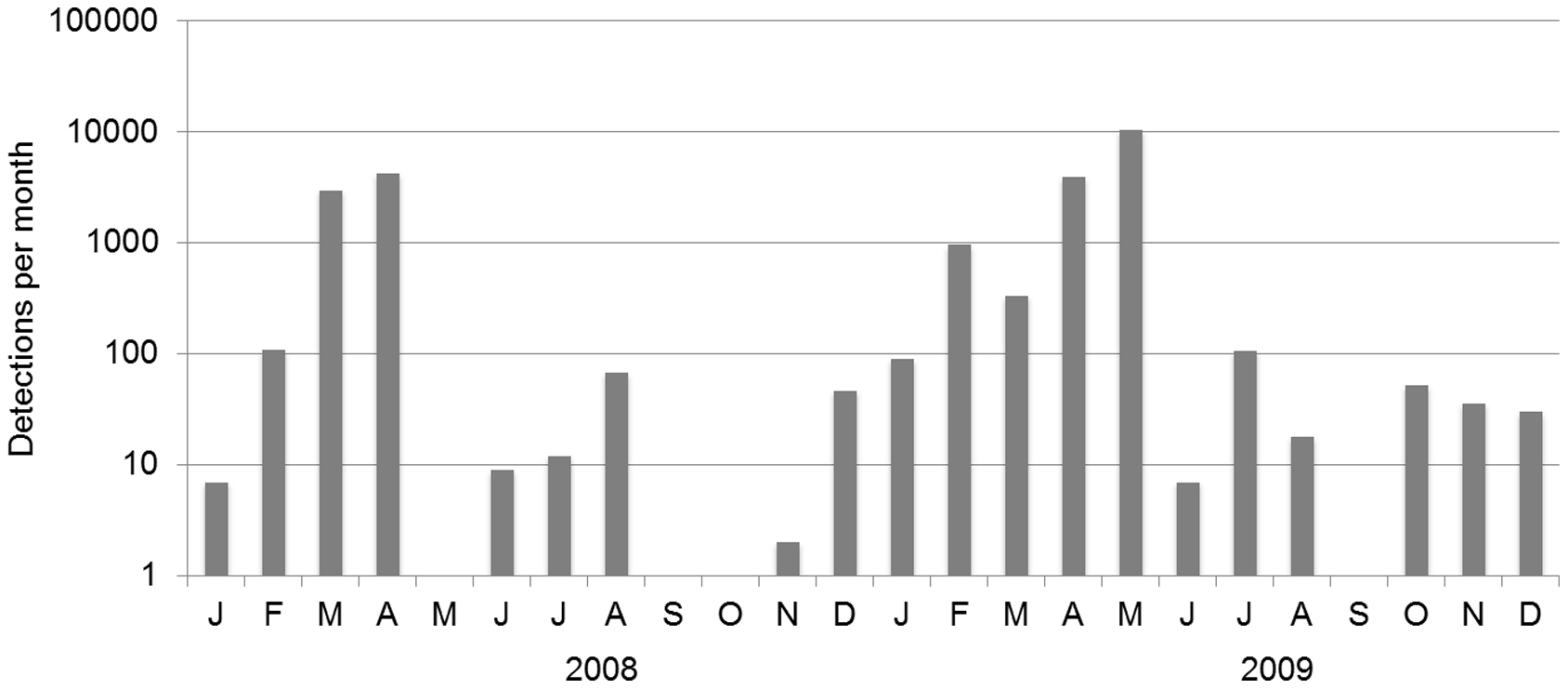
Monthly number of automatically detected and manually verified humpback whale moans for 2008 and 2009. Note that the number of detections is shown on a log scale on the y-axis.

Humpback whale moans were present during 9 months of the year in 2008: January through April, June through August and November and December and 11 months in 2009 (no moans were detected in September; Fig. 4). Typically, moans occurred in bouts, ranging from 2 to 42 consecutive days. For both years, February, March and April had the highest daily occurrence of humpback moans. In May 2009, the daily occurrence of humpback whale moans reached a maximum, whereas in 2008 no calls were present in this month.

### Ice Cover

**Figure 5:**
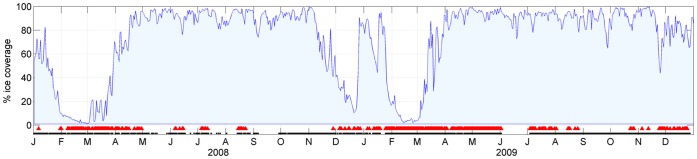
Presence of humpback whale moans in relation to percentage ice coverage off PALAOA. The area graph shows the percentage of ice cover within a 100 km range off PALAOA. Red triangles indicate acoustic presence of humpback whales. Recording status of PALAOA is indicated by the black bar below the acoustic presence indicators (PALAOA is recording = black bar present).

Moans were estimated to be produced by humpback whales at a distance between 2 and 79 km from the observatory. Figure 5 shows the percentage ice cover for the area within a 100 km radius off PALAOA along with the days on which moans were detected (black triangles) and PALAOA recording status (black bar indicates the observatory was recording).

Moans were present in both years from February when open water dominated the area around PALAOA (less than 10% ice cover) to the end of April when percentage of ice cover increased to more than 70%. Calls were furthermore detected in austral winter when percentage of ice cover on some days exceeded 90%. In 2008, calls were absent in September and October and detected again on 27 November, whereas in 2009, calls were absent throughout September until 22 October.

## Discussion

### Humpback Whale Feeding Grounds Near the Ice-shelf Edge

**Figure 6:**
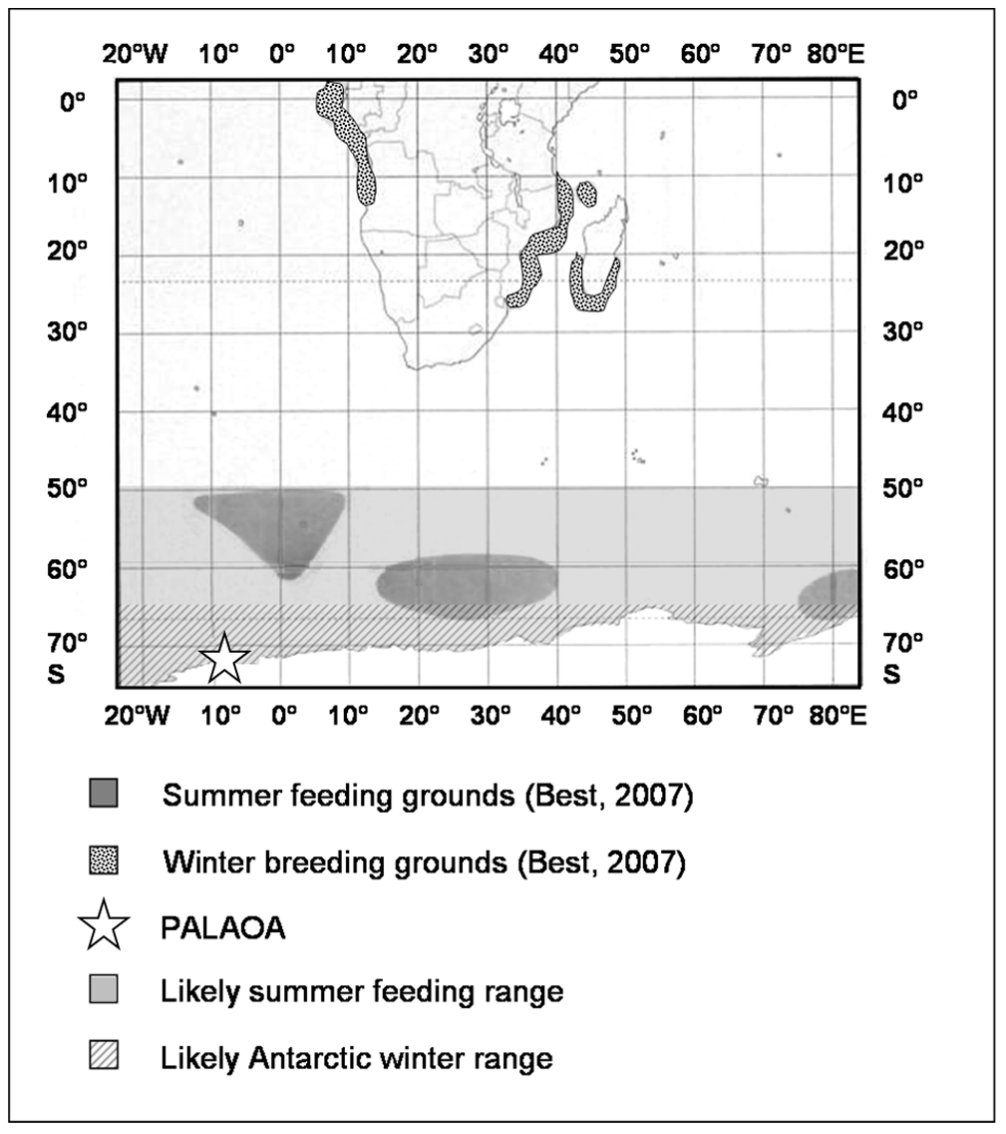
Seasonal humpback whale distribution between 20°W and 80°E. Grey areas indicate regions where summer concentrations are thought to occur for the humpback whale breeding stocks within the Southeast Atlantic and Southwest Indian Oceans [12], [48]. White areas with black dots indicate the winter concentrations. The white star indicates the location of PALAOA. The light grey and the diagonally striped areas represent the larger summer and winter ranges, respectively, as suggested in this study. Figure adapted from P. Best [27]. Reprinted and adapted by permission of the publisher.

The presence of humpback whale vocalizations in the PALAOA recordings from austral summer suggests that humpback whale feeding grounds in IWC areas II and III extend further south than previously assumed [12,27, Fig. 6]. Tynan [28] proposed that humpback whale southbound migration and location of primary feeding areas is linked to the occurrence of predictably productive areas at the southern boundary of the Antarctic Circumpolar Current. With the receding pack-ice in austral summer, whales migrate south of the southern boundary, following the productive marginal ice-edge zone, approaching the continent by February–March [28]. In contrast to more or less confined locations of humpback whale feeding areas as proposed by the IWC [12], Tynan’s [28] and our study suggest that feeding grounds in the Southern Hemisphere are more likely longitudinal regions through which the animals range in a southbound direction while foraging. Humpback whales are known to travel extensive distances on the feeding grounds as part of their foraging strategy [16]. The location of primary feeding areas might therefore not be static, but determined by the interplay of seasonal sea-ice retreat, primary productivity and krill (*Euphausia superba*) abundance. Preliminary analyses of PALAOA data from other years show similar patterns in the presence of humpback whale calls, suggesting that whales return to this area annually.

These observations emphasize the important niche that passive acoustic techniques fill as a method to year round collect data on marine mammal occurrence and behavior in areas such as many parts of the Southern Ocean where access by human observers is (seasonally) restricted.

### Winter Presence

The results of our study show that Southern Hemisphere humpback whales are also present near the ice-shelf edge in the austral winter (Fig. 5). Brown *et al.,* [10] suggested that females might avoid undertaking or completing the long-distance migration each year. Size rather than age is thought to be an important factor determining sexual maturity in humpback whale females [29] and it might therefore primarily be sexually or physically immature females that remain on the feeding grounds all year to maximize growth. These observations along with the relatively large number of Northern Hemisphere humpback whale populations in which individuals have been observed on the feeding grounds in winter [9], [30], [31], [32], [33], suggests that winter presence on the feeding grounds might be common to humpback whale populations in general, and possibly even a general characteristic of baleen whale migratory behavior [34].

The acoustic presence of humpback whales in the region off PALAOA in austral winter (June – August) implies that these whales overwinter in this area. Straley [9] found humpback whales present on Northern Hemisphere feeding grounds throughout winter, although no individual whales overwintered in the feeding area and whales were more likely to be irregular migrants departing late or arriving early on the feeding grounds. In our study, the extent of the Antarctic ice sheet in the mid-winter period with open water mainly occurring in coastal polynyas, is likely to periodically exclude large scale north- or southbound migration of whales. This suggests that whales present on the feeding grounds in winter are more or less confined to these areas until the sea-ice recedes in austral spring.

### Presence in Ice-covered Areas

The acoustic presence of humpback whales in April and in austral winter when sea ice cover in the area around PALAOA is pervasive, contradicts previous suggestions that humpback whales avoid entering ice-covered areas [16], [35], [36]. Little is known about the presence of baleen whales in ice-covered areas, mainly because of the logistic difficulties of accessing these regions, particularly in the Antarctic. Nevertheless, observations of several studies suggest that the availability of polynyas, or areas with open water that reliably occur, rather than the ice itself restricts cetacean distribution [6], [37]. Sirovic *et al.,* [34] used passive acoustic techniques and found blue whale calls present in spring when sea ice cover was still substantial. Minke whales are known to associate with pack ice in winter and autumn [6] and have been observed creating breathing holes in ice [38]. Large groups of various cetacean species were observed being ‘entrapped’ in pools surrounded by vast ice-covered areas in winter [39], [40], [41]. In the Antarctic, the presence of open water and the formation of polynyas are variable and depend on catabatic and westerly winds transporting ice in northern directions [42], whereas easterly winds parallel to the coast transport ice towards the Antarctic continent. Furthermore, the presence of icebergs can affect the formation and size of polynyas which often tend to form on the lee side of (stranded) icebergs or glacier tongues [43]. Variability in the presence and size of areas with open water resulting from ice movements might temporarily limit access to certain areas, possibly explaining the temporal patchiness in the occurrence of humpback whale moans within and between months. In addition, humpback whale movements are likely also affected by krill distribution. In winter, krill is known to prefer under-ice habitat to open water, concentrating near specific sea-ice features such as ridges and polynya borders for feeding and shelter [44]. Although we do not know if humpback whales forage in the area off PALAOA, the coastal polynya likely offers plentiful food supply to humpback whales in winter.

### Conclusions

Our results show that humpback whales may be much more plastic in their migratory behavior than previously assumed, and that many aspects of their migration are still poorly understood [see also 45]. Acoustic recording techniques provide the possibility of monitoring acoustic presence year round and in areas that are (seasonally) difficult to access, but can also be used to gain insights to which breeding stock calling individuals belong [46], [47]. Sounds recorded by PALAOA are likely produced by animals from breeding stock B, off the west-coast of South-Africa, which are thought to migrate to feeding areas between 20°W and 20°E [48]. Several studies have recorded partial and full humpback whale song on feeding grounds [49], [50], [51], which - when compared to recordings from suspected breeding grounds - can provide evidence for stock-specific linkages between areas used for feeding and breeding. Although PALAOA did not record song, humpback whale social vocalizations are often used as units of seasonal song structures [19]. The moans recorded by PALAOA likely lack the required ‘acoustic specificity’, as calls similar to the moan have been recorded in many other areas as well [18], [19], [24]. The humpback whale high call repertoire in the PALAOA recordings, on the other hand, consists of several complexer call types [23] and appears to differ in composition between years (Van Opzeeland, unpublished data). Comparing these high calls to recordings from the various breeding grounds – if available on the appropriate temporal scales - might provide cues to identify to which stock the humpback whales that feed in the waters off PALAOA belong.

Improving the current knowledge on humpback whale migratory behavior is important given that incorrect assumptions on migratory behavior can significantly affect outcomes of population models and management decisions. This is of particular relevance for large baleen species such as humpback whales, which populations were so drastically reduced during the commercial whaling era.
